# Gender-specific differences in haemostatic parameters and their influence on blood loss in bimaxillary surgery

**DOI:** 10.1007/s00784-021-04347-z

**Published:** 2022-01-11

**Authors:** Michael Schwaiger, Sarah-Jayne Edmondson, Jasmin Rabensteiner, Florian Prüller, Thomas Gary, Wolfgang Zemann, Jürgen Wallner

**Affiliations:** 1 Department of Oral and Maxillofacial Surgery, Medical University of Graz, Auenbruggerplatz 5, 8036 Graz, Austria; 2 Department of Plastic and Reconstructive Surgery, Guy’s and St. Thomas’ Hospital, London, UK; 3 Clinical Institute of Medical and Chemical Laboratory Diagnostics, Medical University of Graz, Graz, Austria; 4 Division of Angiology, Medical University of Graz, Graz, Austria

**Keywords:** Orthognathic surgery, Blood loss, Gender, Haemostasis

## Abstract

**Objective:**

The objectives of this prospective cohort study were to establish gender-related differences in blood loss and haemostatic profiles associated with bimaxillary surgery. In addition, we aimed to identify if any gender differences could be established which might help predict blood loss volume.

**Materials and methods:**

Fifty-four patients (22 males; 32 females) undergoing bimaxillary surgery for skeletal dentofacial deformities were eligible for inclusion. Blood samples were taken 1 day preoperatively and 48 h postoperatively for detailed gender-specific coagulation analysis incorporating global coagulation assays (endogenous thrombin potential) and specific coagulation parameters. Blood loss was measured at two different time points: (1) the end of surgery, visible intraoperative blood loss (IOB) using ‘subtraction method’; and (2) 48 h postoperatively perioperative bleeding volume (CBL-48 h) using ‘haemoglobin-balance method’ and Nadler’s formula. Correlation and regression analyses were performed to identify relevant parameters affecting the amount of blood loss.

**Results:**

Significant differences in IOB and CBL-48 h were observed (*p* < 0.001). Men had higher IOB versus women, lacking statistical significance (*p* = 0.056). In contrast, men had significantly higher CLB-48 h (*p* = 0.019). Reduced CBL-48 h was shown to be most closely associated with the level of Antithrombin-III being decreased in females.

**Conclusions:**

Male gender is associated with higher IOB and CBL-48 compared with females. Gender does not affect IOB regarding haemostatic profile but does correlate strongly with procedure length. Conversely, CBL-48 is closely associated with gender-specific imbalances in the anticoagulant system.

**Clinical relevance:**

Knowledge of gender-related differences will help clinicians establish predictive factors regarding excessive blood loss in orthognathic surgery and identify at-risk patients.

## Introduction

While generally considered to be a safe surgical field, orthognathic surgical procedures continue to be linked to large intra- and perioperative bleeding volumes conferring various negative effects for a patient [1–3]. Especially with regard to bimaxillary surgery, involving skeletal repositioning of the upper and lower jaw, blood loss has frequently been described as excessive [4–6]. Reasons for this particularly refer to the rich vascular anatomy of the midface, the limited surgical accessibility to this area, in combination with the complexity of the procedure and the wide surgical exposure needed [6]. Orthognathic surgical procedures are elective by nature; therefore, a high quality of care with minimum associated risks is a necessity.

Against this backdrop, elaborate research into how to prevent and reduce blood loss in this surgical field has been conducted [7, 8]. Throughout the literature, previous studies have also looked into identifying contributing factors which significantly affect blood loss and on the basis of which blood loss may better be predicted [9–12]. In this context, patient gender has frequently been discussed as a major contributing factor in terms of the amount of blood loss to be expected, whereby controversial findings were reported. Several authors have stated that male gender is associated with higher bleeding volumes in comparison to female gender [6, 13–16]. It has previously been suggested that an ampler haemostatic profile in females in comparison with males may trigger gender-specific differences with regard to the bleeding volumes observed [14, 15]. However, the underlying mechanisms potentially affecting blood loss in this regard have scarcely been investigated.

Thus, the aim of this study was to assess blood loss related to bimaxillary surgery with the scientific focus directed towards detecting gender-specific differences. Furthermore, we aimed to identify significant gender-related differences in terms of haemostatic profile and to further link relevant parameters differing significantly to the amount of blood loss detected. As a result, gender-related peculiarities provoking differences in blood loss may be identified and may further be used as predictive factors for the amount of blood loss to be expected.

## Material and methods

This prospective cohort study was conducted at the Department of Oral and Maxillofacial Surgery at the Medical University of Graz in 2019/2020, having obtained approval from the local ethics committee (EK 31–161 ex 18/19).

Male and female patients with skeletal dentofacial deformities scheduled for bimaxillary surgery were selected according to defined inclusion and exclusion criteria. Exclusion criteria entailed (1) additional surgical procedures performed in the same operative session (including genioplasty); (2) oral intake of anticoagulants; (3) coagulopathies; (4) age under 18; (5) cleft lip and palate; (6) connective tissue disorders and (7) ASA grade 3 or 4.

### Perioperative management and surgical protocol

Perioperative treatment followed standardised protocols:

Blood samples were routinely taken on the day prior to surgery on the basis of which a detailed coagulation analysis was performed. Moreover, the level of haemoglobin was determined preoperatively and 48 h after surgery. The patients’ height and weight were measured at admission.

All of the procedures were performed by experienced consultant orthognathic surgeons or advanced surgical trainees under senior supervision, according to standardised surgical protocols. Bilateral sagittal split osteotomy (BSSO) was performed according to Hunsuck and Epker [17, 18], and Le Fort I osteotomy was performed as described by Bell [19].

Intraoperatively, patients were positioned supine with no head-up tilt. Intravenous antibiotics (IV) were given at induction. Total intravenous anaesthesia (TIVA) was used in all patients, with a maintenance target mean arterial pressure of 60 mmHg. Local anaesthesia (Prilocaine 2%, 1: 200.000) was administered intra-orally to the surgical site prior to initial mucosal incision. There was no use of antifibrinolytics or surgical drains. Postoperative patient management included cooling for 2 days, adequate pain control with IV Ibuprofen 600 mg (twice a day on day one and two after surgery) and oral Metamizole and IV Piritramide if required. Patients were nursed at 30°.

Blood transfusions were indicated if the haemoglobin level was less than 6 g/dl, or between 6 and 10 g/dl in cases of cardiorespiratory distress related to excessive blood loss.

### Blood loss

**Fig. 1:**
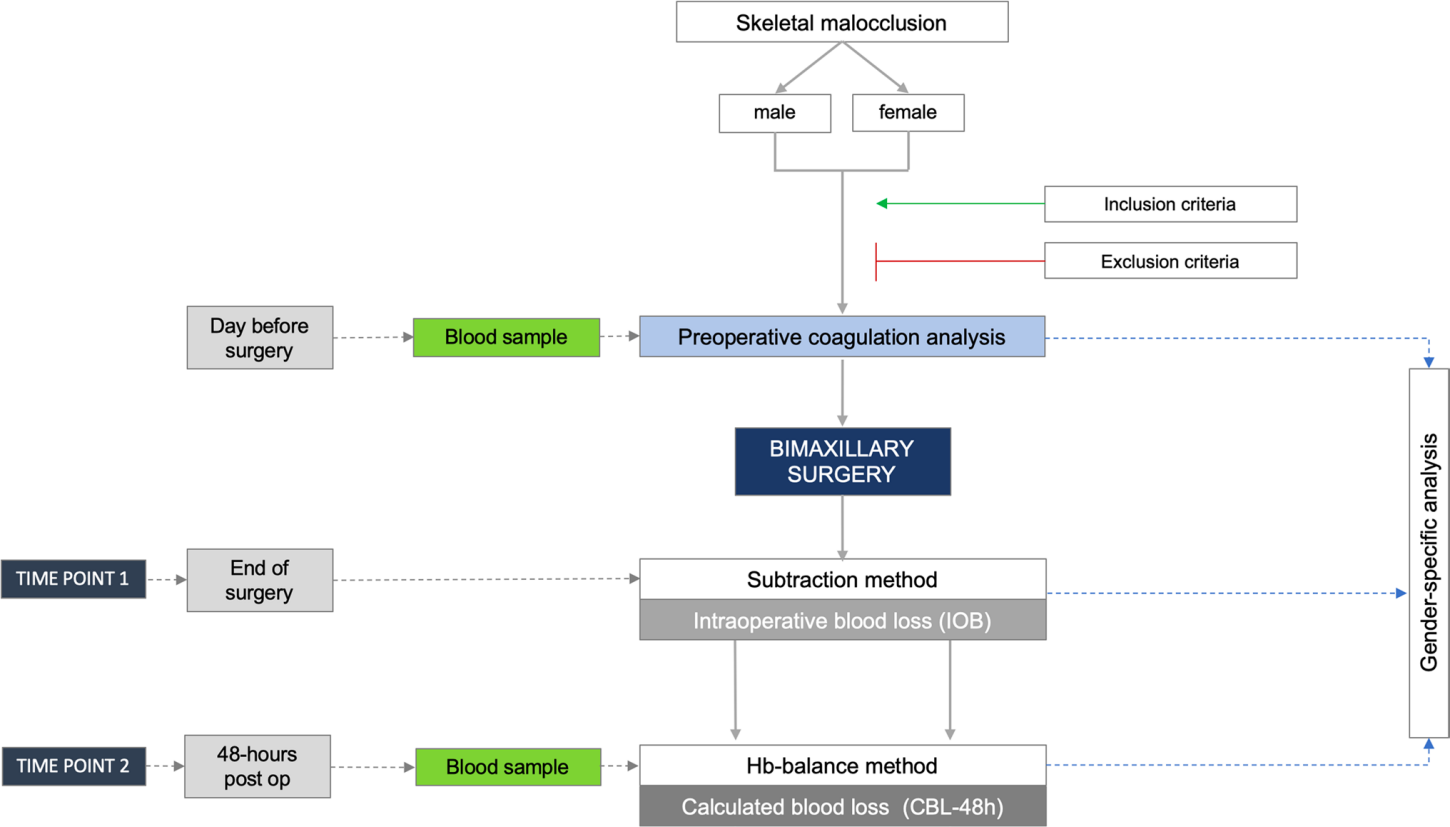
Figure to show the study design of this prospective cohort study

Blood loss was standardly measured at two different time points (Fig. 1).

At the end of surgery, the visible intraoperative blood loss (IOB) was determined consisting of the bleeding volume which had occurred from the time of initial mucosal incision to wound closure. For this purpose, the ‘subtraction method’ was deployed (Table 1): the amount of fluid in the suction canister minus the irrigation fluid used is assumed to be equivalent to the patient’s blood loss [11, 12, 14]. In addition, the weight difference of the throat pack and any surgical gauze used were added to the bleeding volume determined [6, 20, 21].

**Table 1:** The formulae used to calculate blood loss in this present study [6, 22]

Blood loss calculation		
Subtraction method	IOB = fluid in suction cannister − irrigation fluid + weight difference in swabs and throat-pack	IOB = Intraoperative blood loss (ml)
Haemoglobin-balance method	Hb_loss total_ = TBV × (Hb_pre_ − Hb_post_) × 0.001 + Hb_t_ CBL = 1000 × (Hb_loss total_/Hb_pre_)	TBV = total blood volume (ml) Hb = haemoglobin (g/L) t = blood transfusion 1 unit banked blood contains 52 g (± 5.4) haemoglobin CBL = calculated blood loss (ml)

Forty-eight hours postoperatively the perioperative bleeding volume was calculated (CBL-48 h). This was done by means of the ‘haemoglobin-balance method’ relying on pre- and postoperative levels of haemoglobin [22] (Table 1). In addition, this formula requires estimation of a patient’s blood volume total volume, which was established using Nadler’s formula [23] (Table 2).

**Table 2:** Nadler’s formula, which was used to estimate the patient’s total blood volume [23]

Estimated total blood volume (TBV)		
Nadler’s formula	TBV male 0.3669 × H^3^ + 0.03219 × W + 0.6041 TBV female 0.3561 × H^3^ + 0.03308 × W + 0.1833	TBV = total blood volume H = height (m) W = bodyweight (kg)

### Blood parameters and coagulation profile

A detailed analysis of a patient’s haemostatic profile was performed, assessing the following parameters (Table 3):

**Table 3:** Table to show the haemostatic parameters assessed in this study

Preoperative coagulation analysis			
Routine coagulation assays	Global coagulation assay (Thrombin generation assay)		Specific coagulation analysis
aPTT PT INR	Endogenous thrombin potential (%)	ETP_A AUC	Von Wilebrand factor-antigen Von Wilebrand factor-activity Fibrinogen Antithrombin-III Factor VIII Factor IX Factor XI Factor XIII
		ETP_B AUC	
	Peak height of thrombin (%)	ETP_A cmax	
		ETP_B cmax	

#### Routine coagulation assays

##### Activated partial thromboplastin time (aPTT), prothrombin time (PT) and International Normalised Ratio (INR)

These classic coagulation assays allow for basic information on a patient’s haemostatic function in terms of the intrinsic and extrinsic coagulation pathways.

The intrinsic pathway may be monitored using aPTT. Severe to moderate deficiencies of important intrinsic pathway coagulation factors such as II, V, VIII, IX and XI can be detected and usually present with a prolonged aPTT, whereas mild factor deficiencies often remain unnoticed [24, 25].

The prothrombin time (PT) is used to gather information on the extrinsic coagulation pathway, particularly on the coagulation factors II, V, VII and X. The international normalised ratio (INR) has been introduced to improve the comparability of the PT, determined in different laboratories [25, 26].

#### Global coagulation assay

##### Endogenous Thrombin Potential (ETP-auc) and peak thrombin height (ETP-Cmax)

The main task of thrombin is to convert fibrinogen to fibrin, which is an essential step in the haemostatic cascade in order for the blood clot to form [27]. Deficient thrombin formation is associated with excessive bleeding, whereas increased levels of thrombin are linked to thrombosis.

Thrombin formation can be monitored by means of a specific global coagulation assay. This coagulation test generates the so-called thrombogram, which, among others, incorporates the parameters ‘endogenous thrombin potential’ and ‘peak thrombin height’, both of which were assessed in our study [27].

Gender-related differences regarding the aforementioned parameters have frequently been reported, with women presenting with a higher ‘endogenous thrombin potential’ and a greater ‘peak thrombin height’ [28, 29].

##### ETP-auc (%)

The endogenous thrombin potential refers to the net amount of thrombin generated by test plasma under experimental conditions. It is heavily reliant on the balance between procoagulant and anticoagulant parameters. Procoagulant parameters trigger thrombin formation; conversely, anticoagulant parameters inhibit thrombin formation. As a result of this, the ETP allows for detailed information on a patient’s haemostatic function [27].

##### ETP-Cmax (%)

The ‘peak thrombin height’ is defined as the maximum level of thrombin within the thrombogram [27].

#### Specific haemostatic parameters

##### Fibrinogen

Fibrinogen, considered a coagulation factor and a structural protein, is one of the key building blocks in formation of a blood clot. Plasma levels within the normal range are of utmost importance to ensure adequate clot formation and stability. Qualitative or quantitative shortcomings of Fibrinogen present with a variety of symptoms including bleeding; conversely, increased levels of fibrinogen are linked to thrombosis [30].

##### Antithrombin-III (AT-III)

AT-III is a natural anticoagulant, counteracting haemostatic processes. In detail, it inhibits serine proteases such as thrombin, plasmin, kallikrein and coagulation factors IX, X, XI and XII. Patients deficient in AT-III are associated with a severely increased risk of thromboembolism by approximately 50% [31, 32].

##### Von Willebrand factor (vWF)

VWF has several functions within the haemostatic cascade. It is involved in primary haemostatic processes, such as platelet aggregation and adhesion. In addition to that, it serves as a contributing factor within secondary haemostasis by forming a complex with Factor VIII. Deficient or defective vWF are associated with a common bleeding disorder, referred to as von Willebrand disease. Different subtypes of vWD are known, all of which present with typical symptoms such as mucocutaneous bleeding and late-onset haemorrhage [33].

##### Factor VIII (FVIII)

FVIII is part of the intrinsic coagulation cascade and forms a non-covalent complex with the von Willebrand factor. The latter has proven crucial, as it prevents FVIII from premature proteolysis and ensures transportation of FVIII to the site of endothelial injury. Activation of FVIII is established by means of thrombin. Once activated, its purpose is to trigger factor X activation. Decreased activity of FVIII is associated with severe bleeding complications, its intensity depending on the extent of the factor deficiency. While the primary haemostasis works normally in this context, issues arise when it comes to stabilising the blood clot. Congenital factor VIII deficiency is common and is referred to as ‘haemophilia A’ [34].

##### Factor IX (FIX)

FIX plays an important role within the intrinsic coagulation pathway as it activates Factor X in conjunction with FVIII as a contributing factor. Reduced levels of FIX are linked to prolonged bleeding and bruising. Congenital FIX deficiency is known as ‘haemophilia B’ [35].

##### Factor XI (FXI)

Numerous effects of FXI on the intrinsic and extrinsic haemostatic pathway have been described. Most relevantly, it triggers thrombin formation as well as the activation of factor IX and X. Intraoperative haemorrhage has been described in the case of factor XI deficiency; in contrast, increased levels of FXI have been linked to thrombosis [36].

##### Factor XIII (FXIII)

FXIII stabilises the blood clot, making it stiffer and more resistant to fibrinolysis [37, 38]. With regard to decreased plasma levels of FXIII, bleeding complications, especially in the immediate postoperative period, have been found to occur [39–41].

### Statistical analysis

Statistical analysis was performed using IBM SPSS Statistics Version 26 (IBM Corp., Armonk, N.Y.). To statistically assess primary and secondary measures, descriptive statistics together with the following statistical tests were applied: the independent Student’s *t*-test was used for continuous nonparametric data analysis. Correlation between variables was calculated by means of the Pearson Correlation Coefficient. Linear Regression Model was applied to quantify the effect of independent variables on blood loss according to the different time points used and patient gender. A *p*-value of *p* < 0.05 was defined as the cutoff for statistical significance.

## Results

### Patients

Fifty-four patients underwent surgical correction of the skeletal malocclusion in terms of bimaxillary surgery, of whom 22 were males and 32 were females. This accounted for a male-to-female ratio of 1:1.45. The mean age was 27.0 years (± 8.6), with no gender-specific differences detected (*p* = 0.951).

In terms of the body mass index (BMI) determined on the day of admission, men were shown to present with a significantly increased BMI in comparison with women (m: 25.8 ± 4.1; f: 23.3 ± 3.3; *p* = 0.022).

Regarding the ASA status reported, 34/54 patients were categorised as ASA grade 1, whereas 20/54 patients were rated as ASA grade 2. No significant differences between the male and female gender resulted (*p* = 0.582).

### Operation time

The time needed to complete bimaxillary surgery averaged 131 min (± 52.3), taking into account the time from mucosal incision to wound closure.

When analysing the data according to patient gender, the operating time in men was shown to exceed that reported in women by 25.9 min. However, no statistically significant gender-specific differences in terms of the operating time were identified (*p* = 0.076).

### Blood loss

Significant differences in absolute bleeding volumes in terms of the parameters IOB and CBL-48 h were observed (*p* < 0.001). In this regard, a significant increase of blood loss during the first 48 h after surgery was found to occur with IOB amounting to 520.5 ml (± 266.7), and CBL-48 h averaging 802.9 ml (± 275.3).

**Fig. 2:**
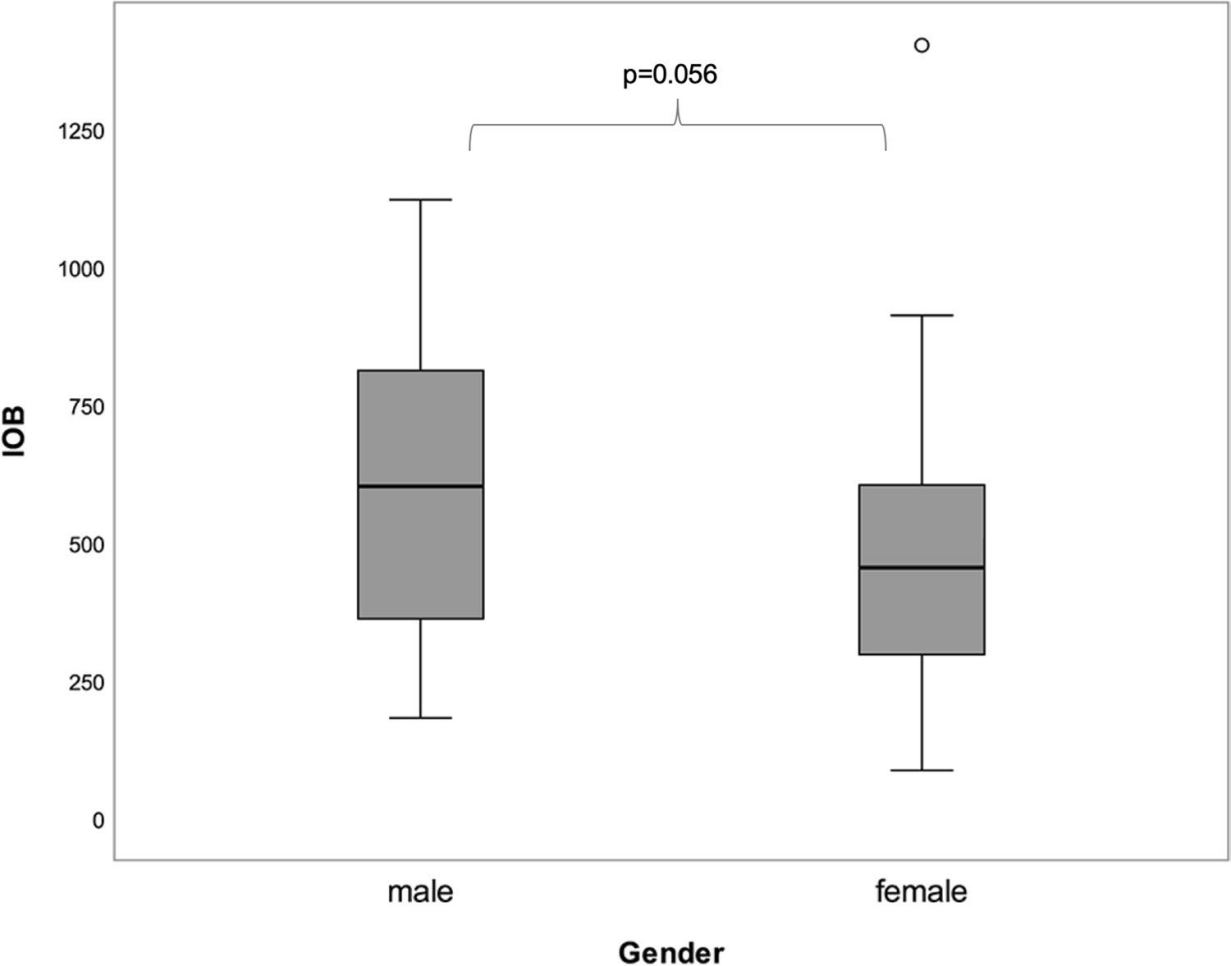
Figure to display the amount of the intraoperative blood loss (IOB) detected according to patient gender. No statistically significant gender-specific differences were found to occur

**Fig. 3:**
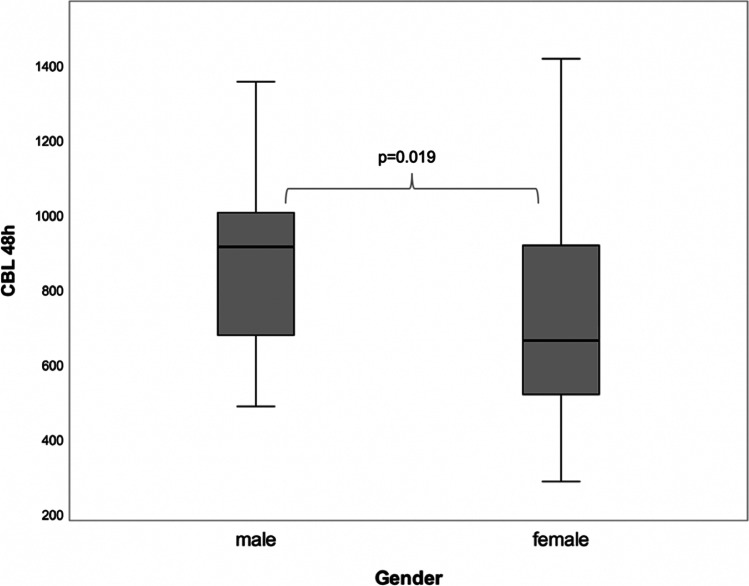
Figure displaying statistically significant gender-specific differences with regard to perioperative blood loss determined 48 h postoperatively (m > f, *p* = 0.019)

Gender-specific subgroup analyses of IOB and CBL-48 h revealed considerable differences among males and females. In terms of IOB, men were shown to present with a higher intraoperative blood loss compared with women, lacking statistical significance (m: 603.9 ml ± 254.8; f: 463.1 ml ± 263.2; *p* = 0.056) (Fig. 2) (Table 4). In contrast, and with reference to CBL-48 h, men were found to be associated with a statistically significantly higher perioperative blood loss than women (m: 907.7 ml ± 246.1; f: 730.8 ml ± 274.5 ml; *p* = 0.019) (Fig. 3) (Table 4).

**Table 4:** Gender-specific analysis of the intra- and perioperative blood loss related to bimaxillary surgery

**Blood loss**						
	**Gender**	**Mean**	**SD**	**Median**	**IQR**	***p*-value**
Intraoperative blood loss (IOB)	m	603.86	254.85	600	455	*p* = 0.056
	f	463.13	263.25	452.5	311	
Calculated blood loss (CBL-48 h)	m	907.73	246.15	912.31	381.17	*p* = 0.019*
	f	730.82	274.54	661.78	407.89

### Gender-related differences regarding blood parameters and the haemostatic profile

#### Blood count

In terms of the blood count analysed prior to surgery, preoperative levels of haemoglobin and haematocrit were shown to differ statistically significantly with respect to patient gender, with men presenting with significantly higher levels in both parameters when compared to women (Hb: *p* < 0.001; Hct: *p* < 0.001). In contrast, the platelet count and the mean platelet volume did not reveal any gender-specific differences.

#### Routine coagulation assays

Analyses of the patients’ haemostatic profile by means of the classic coagulation assays ‘aPTT’, ‘PT’ and ‘INR’ did not show any gender-specific differences in this regard.

#### Endogenous thrombin potential

With reference to the endogenous thrombin potential, monitoring the balance between pro- and anticoagulant parameters by means of thrombin formation and decay, the parameters ‘peak thrombin height’ (ETP-cmax) and ‘area-under the curve’ (ETP-auc) were assessed. No significant gender-related differences in any of the parameters analysed were determined, indicating comparable haemostatic function among males and females (Table 5).

**Table 5:** Gender-specific analysis of the haemostatic profile. Females were shown to present with significantly lower levels of Antithrombin-III when compared with males (*p* = 0.015)

Preoperative coagulation analysis						
	Gender	Mean	SD	Median	IQR	*p*-value
Routine coagulation assays						
aPTT	m	29.25	3.03	28.85	5.85	*p* = 0.102
	f	27.69	3.58	27.7	4.60	
PT	m	101.77	14.16	103.5	17.00	*p* = 0.453
	f	104.37	11.10	103	19.75	
INR	m	0.99	0.09	0.97	0.08	*p* = 0.329
	f	0.97	0.07	0.98	0.09	
Global coagulation assay *Thrombin generation assay* *(ETP)*						
ETP_A_ AUC (%)	m	0.86	0.21	0.89	0.10	*p* = 0.258
	f	0.93	0.17	0.96	0.17	
ETP_B_AUC (%)	m	0.85	0.12	0.85	0.13	*p* = 0.589
	f	0.87	0.18	0.91	0.15	
ETP_A_thrombin peak height (%)	m	1.04	0.24	0.99	0.18	*p* = 0.253
	f	0.96	0.21	0.99	0.19	
ETP_B_thrombin peak height (%)	m	0.96	0.12	0.96	0.21	*p* = 0.753
	f	0.95	0.14	0.99	0.21	
Specific coagulation analysis						
vWF-Ag	m	102.05	38.66	107	72	*p* = 0.198
	f	88.58	33.31	89	52	
vWF-act	m	110.00	76.14	90	99	*p* = 0.064
	f	80.23	34.37	75	43	
Fibrinogen	m	231.47	75.32	204	61	*p* = 0.573
	f	244.06	76.62	237	135	
Antithrombin-III	m	106.11	8.60	106	15	***p* = 0**.015^*^
	f	96.19	15.69	100	22	
FVIII	m	83.61	50.77	69	46	*p* = 0.149
	f	67.20	28.58	65.8	37	
FIX	m	102.76	22.39	97.4	35	*p* = 0.053
	f	90.83	19.46	90.8	24	
FXI	m	97.16	27.75	105	31	*p* = 0.907
	f	98.16	30.28	96	25	
FXIII	m	115.82	27.67	116	53	*p* = 0.385
	f	121.97	23.62	122	45	

### Haemostatic parameters

**Fig. 4:**
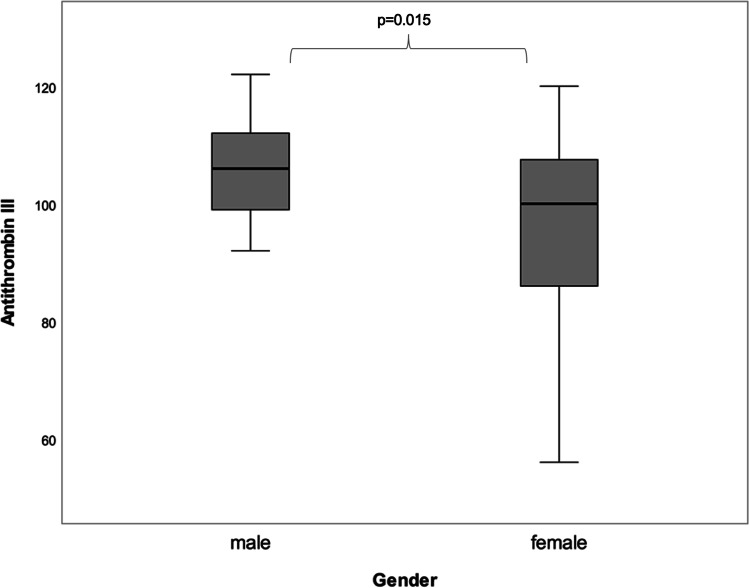
Figure to depict statistically significant gender-specific differences regarding the level of Antithrombin-III measured on the day prior to surgery (m > f, *p* = 0.015)

With regard to the specific analysis of haemostatic parameters prior to surgery, only few of those were shown to differ statistically significantly among the male and female gender. While no gender-specific differences in terms of the parameters Fibrinogen, vWF, VIII, FXI and FXIII were detected, the level ‘Antithrombin-III’ was found to be significantly decreased in women (*p* = 0.015) (Fig. 4). In addition, variations between males and females regarding FIX were observed, lacking statistical significance (*p* = 0.053).

### Correlation analysis

In our study population, the male gender was found to be associated with higher intraoperative and perioperative bleeding volumes in comparison with the female gender (IOB and CBL-48 h). In addition to that relevant gender-specific differences in terms of the natural anticoagulant, Antithrombin-III and coagulation factor IX (FIX) were noted.

To further investigate the effect of these gender-specific differences in terms of the haemostatic profile on the bleeding volumes determined, a correlation analysis was performed.

Furthermore, relevant patient characteristics, such as age, BMI and the ASA status as well the operation time were correlated with the amount of blood loss determined.

The parameters Hb and Hct, differing significantly among males and females, were not considered for this analysis, as this would not have added any value in this context.

#### Antithrombin-III and blood loss

When correlating Antithrombin-III to the amount of intraoperative blood loss detected (IOB), no significant correlations were established. This was also true when further analysing the data according to patient gender.

**Fig. 5:**
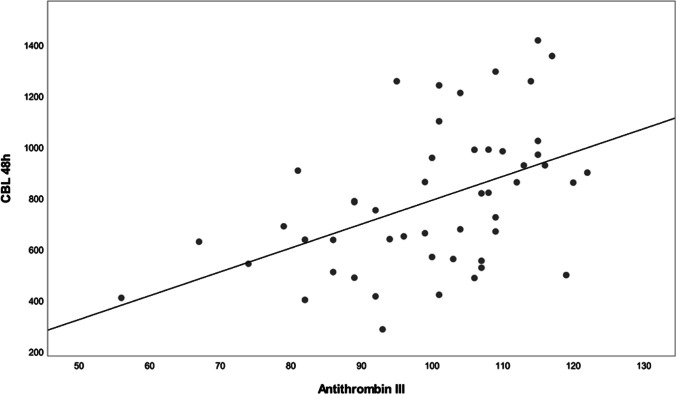
Figure to show the significant positive correlation determined between the level of Antithrombin-III and CBL-48 h (*r* = 0.474; *p* = 0.001)

In contrast, significant positive correlations between Antithrombin-III and CBL-48 h were shown, indicating that the perioperative blood loss increased with the level of Antithrombin-III (*r* = 0.474; *p* = 0.001) (Fig. 5) (Table 6).

**Table 6:** Table to show the statistically significant positive correlations determined in this present study

Correlation analysis		
	IOB	CBL-48 h
Antithrombin-III	*r* = 0.148	*r* = 0.474
	*p* = 0.306	*p* = 0.001*
Operating time	*r* = 0.477	r = 0.307
	*p* = 0.001*	*p* = 0.024*

#### FIX and blood loss

With regard to FIX, no relevant correlations between this specific coagulation parameter and blood loss were found in any of the statistical analyses made.

#### Operation time and blood loss

The operating time was shown to significantly correlate with the amount of IOB and CBL-48 h, indicating that the amount of blood loss determined increased in parallel with the time needed to perform the surgical procedure (Table 6).

#### Age, BMI and ASA status

No significant correlations between any of the aforementioned parameters with IOB and CBL-48 h were found.

### Regression analysis

In a further step, a linear regression analysis was performed taking into account haemostatic parameters differing substantially in terms of gender, as well parameters previously linked to blood loss: patient gender, operation time, age and BMI [6, 12–14, 16].

With reference to IOB (*r*^2^ = 36%), the length of the procedure was found to most significantly affect blood loss (*p* = 0.002). Furthermore, patient age was found to positively correlate with the intraoperative bleeding volume (*p* = 0.33). The haemostatic parameters AT-III and FIX did not correlate with IOB (Table 7).

Regarding CBL-48 h (*r*^2^ = 35%), the level of AT-III was shown to correlate most significantly with the amount of the perioperative blood loss 48 h after surgery (*p* = 0.017). The operating time did not correlate with CBL-48 h (Table 7).

**Table 7:** Linear regression analysis showing that AT-III was most closely associated with the amount of CBL-48 h. In terms of IOB, the operating time was found to be relevant

Linear regression analysis		
	IOB	CBL-48 h
Age	*p* = 0.036*	*p* = 0.991
BMI	*p* = 0.350	*p* = 0.722
Operating time	*p* = 0.002*	*p* = 0.107
Antithrombin-III	*p* = 0.805	*p* = 0.017*
FIX	*p* = 0.496	*p* = 0.772
Gender	*p* = 0.286	*p* = 0.400
ASA-status	*p* = 0.423	*p* = 0.966
*R*^2^	37%	35%

## Discussion

Blood loss and its sequelae remain among the major concerns in the field of orthognathic surgery [1, 42, 43]. Several factors have proven to significantly affect the amount of blood loss with reference to orthognathic surgery, with the length of the operating time together with the surgical modality applied requiring special attention [6, 9, 10, 44]. The patient’s BMI and age have also been contemplated in this context [12, 13].

Patient gender has frequently been proposed as a contributing factor in terms of blood loss in numerous surgical specialties, with the male gender being associated with higher bleeding volumes when compared with the female gender [45–47]. With regard to orthognathic surgery, similar trends have been identified; however, the effect of patient gender on blood loss still remains up for debate. While numerous studies have investigated orthognathic blood loss, only a few of those stated significant gender-specific alterations in this connection [6, 12–16]. All of these studies analysed the amount of the intraoperative blood loss taking into account the blood loss occurring from mucosal incision to wound closure. Olsen et al. exclusively focused on patient gender as the primary predictor variable, monitoring the intraoperative blood loss with regard to bimaxillary surgery [14]. In their study, men were found to bleed twice as much as women (400 ml vs. 200 ml). Significant gender-related differences in terms of the haemostatic profile were held accountable in this context, which will be discussed in detail as this discussion proceeds. While this is striking, it is important to note that the operating time in men exceeded that noted in women by 30 min, lacking statistical significance (*p* = 0.21). In addition to that, sectioning of the maxilla was more frequently performed in the male cohort and no information on the number of genioplasties performed as an adjunct to bimaxillary surgery was provided. Hence, it is pointed out that all of these factors may have accentuated gender-related differences regarding blood loss in their study. Findings reported by Thastum et al. were consistent with those of Olsen et al., stating higher absolute intraoperative bleeding volumes determined in males in comparison with females [12, 14]. Similarly, Rummasak et al. reported higher absolute bleeding volumes in males when compared to females [16]. Secher et al. conducted a randomised controlled clinical trial to investigate the effect of tranexamic acid in a homogenous patient cohort undergoing bimaxillary surgery on patient gender [15]. No significant gender-related differences were identified between males and females in their control group; however, the intraoperative bleeding volume reported in men outreached that in women by approximately 100 ml. Conversely, statistically significant differences in their intervention group were found. In detail, females having received antifibrinolytics were found to bleed significantly less than their male counterparts (367 ml vs. 153 ml). While the length of the procedure was comparable for both groups, no further gender-specific subgroup analysis with respect to the operating time was conducted. Hence, similar to Olsen et al., potential differences in this regard may have biased the bleeding volumes determined [14, 15].

With reference to our study investigating orthognathic blood loss on the basis of a homogeneous patient cohort undergoing highly standardised bimaxillary surgery, relevant gender-related differences between males and females were observed. Regarding IOB, males were found to bleed more in comparison with females by approximately 140 ml (*p* = 0.056).

It needs highlighting at this stage that in addition to the intraoperative blood loss, attention was also paid to the perioperative blood loss occurring within 48 h postoperatively in our study cohort. Statistically significantly higher perioperative bleeding volumes (CBL-48 h) in males were detected in this context (*p* = 0.019) with gender-specific differences averaging more than 170 ml. No comparisons to other studies in this field can be drawn, although, when looking at other surgical specialties, similar findings with reference to perioperative blood loss have been reported. For instance, Hu and Kajja who investigated blood loss related to orthopaedic surgery both suggested significantly increased perioperative bleeding linked to the male gender and, thus, our results align with other research on this topic, showing increased perioperative blood loss associated with the male gender [46, 47].

### Gender-related differences in the haemostatic profile

To investigate the underlying mechanisms triggering these gender-specific differences in terms of the intra- and perioperative bleeding volumes determined in our study cohort, a detailed analysis of the patient’s haemostatic profile was performed on the day prior to surgery. In this context, special emphasis was put on identifying gender-related differences, which might further correlate with the amount of blood loss determined.

In the literature, several blood and haemostatic parameters have been described to differ significantly between male and female subjects; however, only a few such differences were detected in our study cohort [14, 28, 29, 48–50]. It comes as no surprise that the levels of haemoglobin and haematocrit were shown to differ significantly with regard to patient gender, as has also been reported by Olsen et al. [14]. These findings, however, were considered trivial in relation to blood loss and, hence, were excluded from further statistical analyses in our study.

Regarding classic coagulation assays, such as aPTT and PT, which are frequently used within the clinical routine assessment, no gender-specific differences were observed. This, however, does not imply that the haemostatic function in men corresponds exactly to that in women in our cohort. APTT and PT only allow for basic information on a patient’s haemostatic function and are associated with certain limitations [24, 25, 27]. Reasons for this are multifactorial but particularly refer to the design of these assays in combination with the complexity of haemostasis [25, 27]. APTT and PT are considered static coagulation tests and were initially introduced to verify suspicion of bleeding and to monitor the effects of anticoagulant drugs [27, 28]. Thus, their use for the sole purpose of preoperative screening in young and healthy subjects, as was the case in the present study, needs to be viewed rather critically.

With regard to both coagulation assays, fibrin generation is defined as their endpoint. Hence, relevant haemostatic processes, such as the formation of thrombin, are not accounted for. In fact, up to 95% of the thrombin potential remains unnoticed when relying on these tests and as a result of this, haemostatic function and potential cannot be assessed in detail [27, 28].

To adequately monitor the interaction between procoagulant and anticoagulant parameters and to generally get a better understanding of a patient’s haemostatic profile, global coagulation assays need to be deployed. In this present study, a so-called thrombin generation assay was used, which aimed to disclose relevant information about the patient’s haemostatic function [28, 51, 52]. The parameters ‘peak height of thrombin’ and ‘endogenous thrombin potential’, defined as the area under the thrombin curve, were assessed. With regard to the literature, significant differences between males and females have been described on multiple occasions when taking into account the aforementioned parameters [28, 29]. In this context, women have been linked to a greater thrombin potential and a greater peak thrombin height in comparison with men, indicating an ampler, more procoagulant haemostatic profile associated with the female gender.

Among other things, these findings may be explained by potential imbalances with regard to the axis of coagulation factor VIII and protein C, which strongly interrelate with thrombin formation: in females, protein C, which is part of the anticoagulant system counteracting procoagulant parameters, has frequently been shown to be subject to downregulation (i.e. owing to the influence of female sex hormones and the intake of oral contraceptives). This, in turn, leaves factor VIII as the major driver of coagulation, resulting in a more procoagulant haemostatic profile [27, 53]. Hence, it has been hypothesised that gender-related peculiarities related to thrombin formation and decay may be accountable for the significant differences detected among males and females in terms of the bleeding volumes determined. With reference to our study cohort, however, no such differences in terms of the thrombin potential were observed, and hence, this hypothesis was rejected in our study.

Olsen et al. relied on another global coagulation assay, referred to as thromboelastography, with the aid of which a patient’s clotting dynamics were assessed [14, 52]. In this connection, females presented with a more procoagulant haemostatic profile than men in their study [14]. Furthermore, the authors found that the level of fibrinogen differed statistically significantly among males and females and they were further able to entrench a significant correlation between the intraoperative blood loss related to bimaxillary surgery and the aforementioned parameter. The latter appears comprehensible, as adequate clot formation is heavily reliant on Fibrinogen [30]. Women presented with higher levels of Fibrinogen in comparison with men, which was considered co-responsible for reduced intraoperative bleeding associated with the female gender. Their study population was, however, small and included only 15 women. At the same time, similar gender-related differences with regard to Fibrinogen in a study with more than 300 patients were observed, which support the findings of Olsen et al. [14, 50]. In contrast to these findings, no gender-related differences regarding fibrinogen resulted in our study.

Speaking of statistically relevant gender-related differences in terms of the haemostatic profile, these were confined to the levels of Antithrombin-III in our study (*p* = 0.015). Regarding this specific anticoagulant parameter, decreased levels in females in comparison with men were observed. When further reflecting on these differences and contemplating the function of AT-III within the haemostatic cascade, it seems likely that a reduced activity of this parameter in females could have triggered a decrease in blood loss [32].

To prove this hypothesis, the anticoagulant parameter AT-III as well as parameters previously described to affect blood loss, such as the operating time and age, were correlated with IOB and CBL-48 h [6, 12]. Surprisingly, no effects of gender-based peculiarities in terms of the haemostatic profile on IOB were observed. This is in marked contrast to the findings reported by Olsen et al. who stated a significant correlation between IOB and an ampler haemostatic profile [14]. As opposed to this, a significant correlation between decreased levels of AT-III and reduced perioperative blood loss (CBL-48 h) was established in our study.

In terms of the operating time, which is indeed an important parameter to consider, the IOB was shown to highly significantly correlate with the length of the procedure; intriguingly, a weaker effect on perioperative bleeding was observed in this regard. Conclusions drawn from a linear regression analysis regarding contributing factors significantly influencing the intra- and perioperative blood loss were consistent with those from the correlation analysis, whereby no effect of the operating time on CBL-48 h was observed. What is more, age was found to affect the IOB in this analysis; however, these findings were shown to coincide with a longer operating time in older patients.

Considering the findings related to AT-III, we hypothesised that IOB was unaffected by gender-related differences, as the full pro- and anticoagulant potential might only be expressed after wound closure. This implies that imbalances with regard to the haemostatic profile may only become evident at a later stage, which will most likely be reflected in the amount of perioperative blood loss.

Several important clinical implications result from these findings, which specifically include adequate preoperative screening of haematological parameters to minimise the risk of excessive blood loss and associated side effects. While numerous guidelines are available with the aid of which suspected bleeding can be assessed [54, 55], these do not apply in the present context, as patients going for orthognathic surgery are generally young and healthy and do not show any signs of increased blood loss beforehand. In contrast and as already mentioned earlier in the manuscript, the sole use of aPTT and PT has also proven contentious owing to the shortcomings associated with these coagulation assays. More specific coagulation analysis may therefore be considered necessary to identify patients at risk of increased blood loss. Global coagulation assays allow for detailed information about the patient’s clotting dynamics; however, these may not be available within the clinical routine assessment [52]. Against this backdrop, it is suggested to additionally consider analysis of more specific coagulation parameters, such as Fibrinogen and Antithrombin-III, which have already been found to affect blood loss in orthognathic surgery [14]. Furthermore, those parameters should be taken into account, which have commonly been associated with bleeding disorders. As a result of a more detailed coagulation analysis, patient safety may be improved and a more targeted use of antifibrinolytics may be entrenched. Especially with regard to the female gender, where a more procoagulant profile appears to decrease blood loss, and, in combination with specific patient characteristics such as the intake of oral contraceptives and smoking, which may potentially increase the risk of thrombosis, these substances should only be used in cases where increased blood loss is to be suspected.

Limitations of this study include the fact that determination of blood loss is still based on approximation and, thus, the bleeding volumes reported may not be entirely accurate. The subtraction method, applied to monitor the intraoperative blood loss, has frequently been shown to underestimate the actual bleeding volume [6, 21, 56]. This is thought to be due in part to bleeding into tissue spaces, such as the maxillary sinuses, which is therefore not accounted for [6]. Regarding the perioperative blood loss, the formula used to calculate blood loss in conjunction with the time-point chosen has been shown to strongly influence the bleeding volumes determined [57]. While we acknowledge these potential shortcomings, it has previously been shown by our group that the formula applied does not significantly alter results, when measuring blood loss 48 h after surgery, where normalised blood levels are to be assumed. To additionally mitigate the risk of the formulae used influencing outcomes in our cohort, we relied on a highly-standardised perioperative protocol ensuring that the blood samples required were taken at the ordered time points, that none of the patients was fasted at the time of the preoperative blood sample and that the use of postoperative IV fluids was limited to the immediate postoperative period (6–8 h postoperatively). As a result, we propose that the risk of blood dilution and differing time-points potentially tampering our results was reduced to a minimum. Regarding the IOB, we suggest that considering the weight difference in swabs and surgical gauze additionally added to the validity of our results.

Further limitations of this study may refer to the fairly small sample size, which, in turn, could have affected the power of the disclosed results. However, in comparison to other research on gender-specific blood loss in orthognathic surgery, this study population represents one of the biggest cohorts investigated as of yet. What is more, this study was conducted prospectively on the basis of well-defined inclusion and exclusion criteria, highly standardised surgical protocols and multiple perioperative measures and also included a detailed coagulation analysis. Still, we appreciate that a larger patient cohort, assessed according to the same scientific standards as in the present study may have potentially increased the power of our study.

## Conclusion

To conclude, the amount of the intraoperative blood loss (IOB) determined remained unaffected by gender-based differences in terms of the haemostatic profile but was shown to strongly correlate with the length of the procedure. Conversely, the amount of perioperative blood loss, which differed significantly among males and females, was found to be most closely associated with gender-specific imbalances in terms of the anticoagulant system. This implies that gender-specific differences regarding blood loss in orthognathic surgery mainly became apparent in the immediate postoperative period.

Further prospective studies and detailed research into haemostasis and blood loss in the field of orthognathic surgery are still needed to better understand underlying mechanisms related to our findings. This will help to establish gender-specific predictive factors regarding excessive blood loss in orthognathic surgery and identify at-risk patients. In addition, this will allow accurate preoperative screening and optimise the use of perioperative measures, such as tranexamic acid, to reduce and prevent blood loss where indicated.
